# A Tale of Two Epidemics. March 20, 2020

**DOI:** 10.1017/dmp.2020.58

**Published:** 2020-12

**Authors:** James J. James

We are all too aware of the first epidemic—over 120,000 cases reported from well over 100 countries with some 4,500 deaths and another 6,000+ severe or critical cases. Large outbreaks have occurred, and local medical facilities overwhelmed, with many hospitals literally turned into war zones with medical personnel surrounded by suffering and death and having little at their disposal to combat the microscopic foe. From the perspective of the individual healthcare worker, trained in the ethos of individual care, this is a real and harrowing experience; for those not yet infected such images, well publicized in traditional and social media, naturally lead to feelings of concern and vulnerability. With widespread concern and alarm governments activate measures in an attempt to contain the epidemic. Such measures include quarantines, isolation, and travel restrictions in an effort to prevent introduction of the causative agent and/or its spread. When it becomes obvious that such measures are only partially effective and an ever-increasing number of cases are reported from more locations, legitimate concerns evolve into fear and panic which are constantly refueled by media that seem to be in constant competition to outdo each other with dramatic, sensationalized headlines. And thus, we have a second epidemic of COVID-19, one largely of our own creation, that has truly evolved into national epidemics potentially far more destructive than the first: Population Panic, or relentless fear that leads to an excessive response that is difficult to justify given the actual risk involved.

In justifying this diagnosis, we first need to look at the data and information we have to date on the clinical epidemic and try to estimate the risk; what is the approximate risk for individuals to come in contact with the COVID-19 virus and, given exposure, the risk of illness and death. These numbers are, first and foremost, estimates based on data to date and will change as the clinical epidemic evolves and we understand more. However, at this point in time, given the large number of cases, any estimate should be a reasonable approximation of what to expect.

The two defining parameters for the severity of an epidemic are the number of people infected and the case mortality rate. If we look at Hubei province in China alone, the epicenter of the clinical epidemic with a population of some 15 million, and ascribe all cases and deaths to that area, we come up with a population attack rate of .8% and a mortality rate of .02%, a far cry from the corresponding estimated rates of 30% and 2.5% for the 1918 influenza epidemic, which has become the benchmark measure of a modern pandemic. Thus, from a population perspective, the risk of clinical illness and/or death is comparatively low. What is more meaningful in terms of COVID-19, is the case mortality, a gross estimate of which is 3.5%. However, this number is somewhat misleading in two ways: (1) risk of death is not evenly distributed across age groups, and, if you look at age-adjusted mortality, any relatively healthy person under 60 has very little risk of a fatal event and children seem to be practically immune; (2) we are using the term “case” to identify both those who have clinical symptoms, and those who have tested positive but are asymptomatic. This latter group is an extremely important one to keep in mind as they certainly must be considered capable of transmitting the virus. From the Diamond Princess screening we can estimate that 50% of COVID-19 cases are asymptomatic carriers. Broader population testing would be required to determine if this figure is much higher.

This, of course, brings up the issue of transmission, where many questions remain. Most importantly to consider, however, is that this is a respiratory virus and the primary mode of human-to-human spread is respiratory droplets. The role of other mechanisms, especially through hand contamination, are important, but would be expected to be secondary to inhaled droplets. The real unanswered question is the danger inherent in exposure to sub-clinical cases and the prevalence of those cases. As to the latter, we have to accept that virtually millions of travelers traveled to and from Hubei province, many by international air between December 8, 2019, when the first symptomatic case was identified, and January 23, 2020, when the Chinese quarantine was imposed. One can only conclude that literally every region across the globe has already been exposed to some extent and travel restrictions may have less than the desired effect on preventing exposure. The effectiveness of quarantine is always debated but that imposed in China seems to have had the desired effect.

Overall, taking an objective view of the data and knowledge we have to date should lead us to some sobering conclusions. The COVID-19 clinical epidemic is here, we are not going to keep it out; cluster outbreaks can be devastating to affected communities and their health systems, but we must expect them; this epidemic is not going to fade away, it is already too deeply rooted in the global ecosystem, and may well join Tuberculosis, HIV, Malaria and a host of other infectious maladies that significantly add to our collective morbidity and mortality. We have learned to deal with or accommodate these other diseases and we must learn to do the same here while we await the development of vaccines and other pharmacological countermeasures. What we must be careful of is an excessive reaction, as if this were a species-ending event—it is not. It is, in fact, far less deadly than many other infectious disease scourges that we abide every day without imploding the economy and potentially ushering in a host of dire social consequences far more damaging than COVID-19.

In my opinion, from a U.S. perspective, we have to move forward and begin to focus our attention on what we know and not what might be. Most importantly, we have to end the blame game between and within countries and cease stigmatizing individuals and ethnic groups. The Population Panic may be self-induced but COVID-19 is a work of nature and a true Trojan Horse. In addition, at the national level we need to stop politicizing a true public health emergency. As the COVID-19 epidemic evolves we will need bi-partisan support to craft the necessary legislation to support such state-level initiatives as school closings and business curtailments, as well as the provision of resources to better enable our medical and public health systems and give relief to existing legal constraints on privacy and inter-state commerce. Further, the most critical infrastructure that we have going forward is the health care system, and simultaneously the one at greatest risk. Lessons learned from medical centers overseas must be kept in mind and appropriate policies and protocols put in place with plans for relief of those that become epicenters of area outbreaks. At the community and individual levels we need to strongly encourage social distancing and the good hygiene measures we have had ingrained; most importantly, clinically symptomatic individuals should be aggressively identified, evaluated, diagnosed, and those with COVID-19 receive the appropriate medical and public heath interventions. Most importantly, we have to do everything possible to protect our seniors from exposure to symptomatic cases by all and whatever means are available. Finally, we have to address the Population Panic and here we need the cooperation and commitment of the media as part of the health care team. Instead of fear-sustaining headlines on worst case scenarios and system failures, we need to educate the public, especially the vast majority of those under 65 (approximately 85% of our population), that for them this is not a life-threatening infection. If in retrospect we have to say the operation was a success but the patient died, then none of us will have done our job.

